# Influence of seasonal and climatic variables on coffee berry borer (*Hypothenemus hampei* Ferrari) flight activity in Hawaii

**DOI:** 10.1371/journal.pone.0257861

**Published:** 2021-12-20

**Authors:** Melissa A. Johnson, Nicholas C. Manoukis

**Affiliations:** Daniel K. Inouye US Pacific Basin Agricultural Research Center, United States Department of Agriculture–Agricultural Research Service, Hilo, Hawaii, United States of America; US Department of Agriculture, UNITED STATES

## Abstract

Coffee berry borer (CBB, *Hypothenemus hampei* Ferrari) is the most serious insect pest of coffee worldwide, yet little is known about the effect that weather variables have on CBB flight activity. We sampled flying female CBB adults bi-weekly over a three-year period using red funnel traps baited with an alcohol lure at 14 commercial coffee farms on Hawaii Island to characterize seasonal phenology and the relationship between flight activity and five weather variables. We captured almost 5 million scolytid beetles during the sampling period, with 81–93% of the trap catch comprised of CBB. Of the captured non-target beetles, the majority were tropical nut borer, black twig borer and a species of *Cryphalus*. Two major flight events were consistent across all three years: an initial emergence from January-April that coincided with early fruit development and a second flight during the harvest season from September-December. A generalized additive mixed model (GAMM) revealed that mean daily air temperature had a highly significant positive correlation with CBB flight; most flight events occurred between 20–26°C. Mean daily solar radiation also had a significant positive relationship with flight. Flight was positively correlated with maximum daily relative humidity at values below ~94%, and cumulative rainfall up to 100 mm; flight was also positively correlated with maximum daily wind speeds up to ~2.5 m/s, after which activity declined. Our findings provide important insight into CBB flight patterns across a highly variable landscape and can serve as a starting point for the development of flight prediction models.

## Introduction

Coffee berry borer, *Hypothenemus hampei* (Ferrari) (Coleoptera: Curculionidae) is the most damaging insect pest of coffee worldwide, causing more than $500M in annual crop losses [1]. The female coffee berry borer (CBB) initiates infestation when she bores an entrance hole into the coffee fruit (“berry”) and builds galleries for reproduction in the seed (“bean”). The offspring feed on the endosperm tissue, causing further damage to the bean and resulting in reduced quality and yields [1, 2]. Managing CBB is particularly difficult due to the cryptic nature of its life cycle which occurs almost entirely within the coffee berry [3, 4]. Male and female siblings mate within their natal berry, the males die, and mated females leave in search of a new berry to infest [1]. This is the time when adult CBB are most vulnerable to chemical and biological controls [4, 5].

Integrated pest management (IPM) of CBB typically involves multiple components including sprays of *Beauveria bassiana* (an entomopathogenic fungus) early in the season, frequent and efficient harvesting, and post-harvest sanitation [3, 5, 6]; these have been shown to be effective for managing CBB in Hawaii [7, 8]. A critical aspect of most successful IPM programs is monitoring of pest activity with traps and/or infestation assessments. By identifying peaks in flight activity that coincide with berry colonization, coffee growers can maximize the efficiency of *B*. *bassiana* applications and thereby minimize costs. Traps are used in many countries to monitor CBB activity [9–12] and mass-trapping using a high density of traps in an area has even been implemented as a possible method of control [13]. In Hawaii, traps were introduced soon after the initial CBB detection in 2010 [14] as part of the IPM guidelines for managing this new invasive pest [15]. Traps were adopted by many growers in the early years of the invasion to monitor CBB activity in their fields and time pesticide sprays. However, over time, fewer and fewer growers utilized traps with most transitioning to calendar sprays of *B*. *bassiana* or relying on casual observation of infestation in their fields to guide the timing of spray applications (A. Kawabata, pers. comm.). This approach has been shown to be inefficient: Hollingsworth et al. [7] reported that 3–5 sprays of *B*. *bassiana* conducted early in the season (based on information from monitoring) were as effective as 8–12 calendar sprays, highlighting the importance of monitoring flight activity to appropriately time sprays for cost-effective control of CBB.

Understanding the seasonal phenology of CBB and the underlying abiotic drivers of flight are essential for predicting periods of high activity. Information on CBB population dynamics for a given coffee-growing region can be used to develop action thresholds and forecasting models for specific locations. This is especially important for Hawaii where the coffee-growing landscape is heterogeneous, and a single set of management recommendations is difficult to apply across the entire region. Coffee is grown commercially on six of the main Hawaiian Islands on volcanic soils that vary in age and nutrient composition and experience a broad range of microclimate conditions from sea level to over 800 m in elevation [16]. On Hawaii Island, more than 800 small farms produce coffee, most of which are family-run operations that rely on manual labor to harvest the coffee and implement management practices. Optimizing pest management strategies while minimizing costs are critical to the longevity of these farms as Hawaii has some of the highest labor and production costs of any coffee-growing region.

In the present study we monitored the flight activity of CBB via trap catch at 14 commercial coffee farms in the two main coffee-growing regions of Hawaii Island, Kona and Kaʻu, over a three-year period. Five weather variables thought to be important for insect flight (temperature, relative humidity, solar radiation, wind speed and rainfall) were tracked at each site. Our objective was to describe the seasonal phenology of CBB across a highly variable landscape, as well as to observe abiotic factors that may trigger CBB flight, which will aid in the development of models for predicting future flight events. These models can serve as decision support tools to guide the timing of pesticide applications. While this study is focused on Hawaii, we expect that the information gained on CBB flight and associated weather variables will be useful for developing IPM strategies in other coffee-growing regions.

## Materials and methods

### Study sites

**Fig 1:**
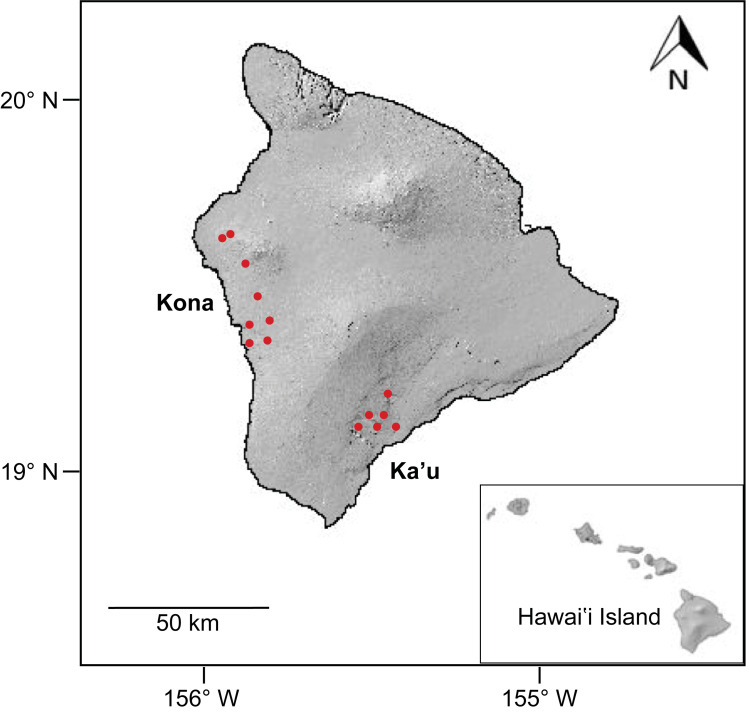
Map of Hawaii Island showing 14 study sites (eight in the Kona district and six in the Kaʻu district). Inset map shows the main Hawaiian Islands and the location of Hawaii Island within the archipelago.

Fourteen commercial coffee farms were selected for the study on Hawaii Island. Eight farms were located on the West side of the island in the Kona district and six farms were on the Southeast side of the island in the Kaʻu district (Fig 1). While coffee is grown throughout the Hawaiian Islands, Kona and Kaʻu are two primary coffee-growing regions, both of which are world-renowned for the high quality of their coffee. Farms were selected to encompass the broad range of elevations and climatic conditions under which coffee is grown on Hawaii Island. In Kona, farms ranged in elevation from 204 m– 607 m and in Kaʻu farms ranged from 279 m– 778 m in elevation. All farms were actively managed for coffee production throughout the study, although the timing and number of interventions varied. Management practices included regular pruning, weed management, fertilizer and pesticide application, cherry harvesting and end of season strip-picking. Farms were largely characterized as sun-grown although some farms had scattered fruit, nut or ornametal shade trees planted as well. All farms had *Coffea arabica* var. *typica* planted with the exception of one farm in Kaʻu which had primarily var. *catuai* planted. Permission was obtained from each private land owner prior to initiating the study.

### Flight activity

Red funnel traps (CIRAD, Montpellier, France) baited with an alcohol lure (3:1 methanol:ethanol) were haphazardly distributed throughout each farm. Trap density was based on farm size, with 3–5 traps used for small farms (1–1.4 ha) and 6–9 traps used for large farms (1.5–2 ha). Traps were hung on stakes at ~1 m in height and were equipped with a collection cup containing propylene glycol. Trap contents were collected in 70% ethanol on a bi-weekly schedule from 2016–2018. Lures were refilled as needed and propylene glycol was replaced bi-weekly. In the laboratory, trap contents were passed through a sieve (1.5 mm mesh size) to seperate out all large insects, which were discarded. The remaining insects from each trap collection were placed under a stereomicroscope (Leica microsystems GmbH, Wetzlar, Germany). All scolytine beetles were counted and CBB were seperated from these other beetles. If >500 beetles were caught in a single trap, we used a volumetric method to estimate count (see [17] for additional details on trap setup, collection and processing). The number of CBB and other scolytids caught per trap per day (average of 14-day intervals over the study) was calculated for each site.

### Weather variables

Manual or cell-service weather stations were set up at each farm to measure the following variables: air temperature, relative humidity (RH), rainfall, wind speed and solar radiation. Manual stations consisted of a Hobo Pro v2 temperature/RH data logger (U23-002, Onset Computer Corporation, Bourne MA) housed in a solar shield (RS3, Onset Computer Corporation, Bourne MA), a solar pendant (UA-002-64, Onset Computer Corporation, Bourne MA) and a rain gauge equipped with a manual data logger (RainLog 2.0, RainWise Inc.). Cell-service weather stations were comprised of a 4G remote monitoring station (RX3004-00-01, Onset Computer Corporation, Bourne MA) equipped with a temperature/RH sensor (S-THB-M002), solar panel (SOLAR-5W), solar radiation sensor (S-LIB-M003) placed within a solar shield (RS3-B) and rain gauge (S-RGB-M002). Wind speed sensors (S-WSET-B) were added to each cell-service station in 2018. Sensors were located between 1–3 m above the ground on each weather station, depending on the type of sensor. For each site, the daily maximum, mean, and minimum were estimated for air temperature and RH in R v. 3.5.0 using the ‘aggregate’ function in the *stats* package [18]. We used the same method to estimate the daily mean and maximum wind speed and solar radiation, as well as daily cumulative rainfall. We then used these daily values to calculate the average air temperature (°C), RH (%), wind speed (m/s) and solar radiation (W m^-2^), as well as the cumulative rainfall (mm) for each ~bi-weekly sampling period.

### Data analysis

All statistical analyses were conducted in R v.3.5.0 [18]. The assumption of normality for each variable was assessed using quantile-quantile plots and a Shapiro-Wilks test; an *F* test was conducted to check for equal variances using the *stats* package. A Pearson correlation test was conducted using the *stats* package to examine the relationship between total CBB capture for each year/site and elevation. The mean number of CBB caught per trap per day was log-transformed (log + 1) prior to analysis. Linearity of the relationship between CBB/trap/day and each weather variable was also checked prior to analysis using two-dimensional scatterplots. A principal components analysis (PCA) was first conducted on the full set of environmental variables and the log-transformed mean number of CBB/trap/day for the 14 sites. Given that complete data sets are needed to conduct PCA and we had some gaps in our weather data (17% missing) due to sensor malfunctions and the late addition of wind sensors to the study, we opted to fill gaps using data collected either 1) on the day/s in question but from neighboring sites if available, or 2) on the day/s in question but in subsequent years (2019–2020) at the same site.

The influence of weather on CBB flight activity was then evaluated with a generalized additive mixed model (GAMM) in the *mgcv* package v. 1.8–23 [19], which we deemed appropriate given the non-linear nature of the relationship between weather variables and CBB flight. The response variable for the GAMM model was the log-transformed mean number of CBB/trap/day, with year and site included as random effects and the weather variables included as fixed effects with cubic regression splines. We assumed a Gaussian error distribution and an identity link function for the model. We used the generalized cross-validation (GCV) score to measure model smoothness with respect to the smoothing parameters as well as the estimate prediction error. A lower GCV score indicates a smoother model and is somewhat comparable to an Akaike Information Criterion (AIC) value, in that a lower score equates to a better fitting model. Pairwise Pearson correlation tests of continuous explanatory variables were conducted to assess multicollinearity at a correlation coefficient threshold of 0.7; maximum temperature, mean RH and maximum solar radiation were subsequently dropped from the model due strong correlations with mean temperature, maximum RH and mean solar radiation, respectively.

## Results

### Trap catch

**Fig 2:**
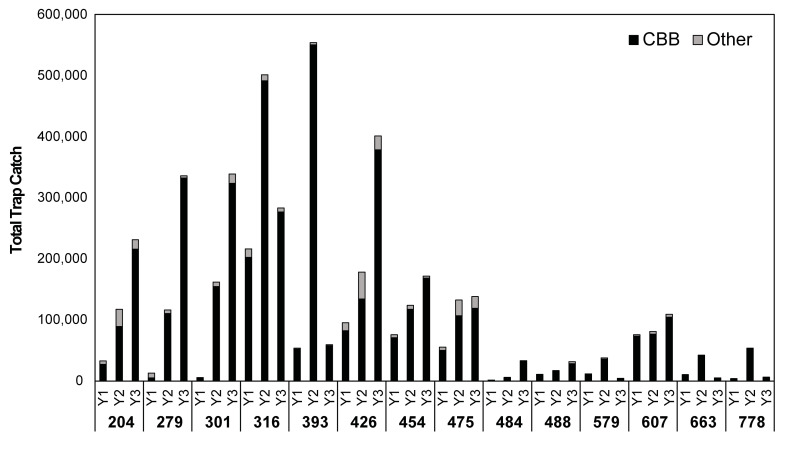
Total trap catch per site across each of three years (Y1: 2016, Y2: 2017, Y3: 2018) on Hawaii Island. Study sites are in order of increasing elevation from left to right. Black bars represent coffee berry borer (CBB) while gray bars represent other scolytid beetles.

In total, just under 5 million scolytid beetles were captured over a period of 143 weeks. Across all 14 sites, CBB made up an average of 81% of the total trap catch in 2016, 91% in 2017, and 93% in 2018 (Fig 2). The most trapped non-CBB beetles were *Hypothenemus obscurus* F. (tropical nut borer), *Xylosandrus compactus* Eich. (black twig borer) and a species of bark beetle in the genus *Cryphalus* Erichson. Peak activity for non-CBB beetles was in the summer months of June and July. Farms observed to have higher percentages of non-CBB beetles in traps were located next to macadamia nut orchards or forests or had other fruit and nut trees interplanted with the coffee.

### Seasonal phenology of CBB

**Fig 3:**
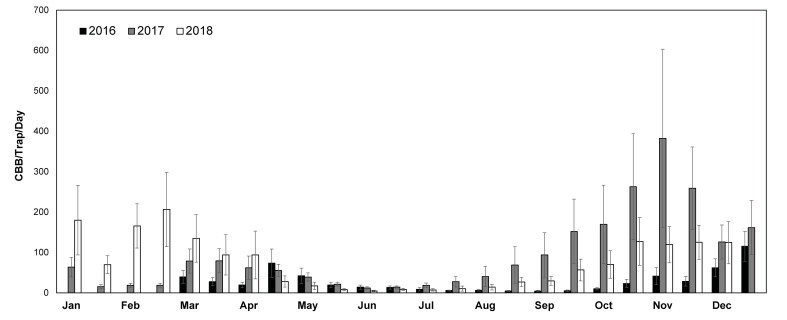
Seasonal flight phenology of coffee berry borer (CBB) on Hawaii Island over a three-year period. Sampling began in March (Kona) or May (Kaʻu) 2016 and ended in December 2018. Error bars show the variation across 14 study sites.

The combined CBB catch across all sites was similar for 2017 (~1.98M CBB) and 2018 (~2.05M CBB), but considerably lower for 2016 (~600K CBB). This is likely because data collection did not start until March (Kona) and May (Kaʻu) in 2016, so numbers from the initial emergence are missing for that year. Although variation was observed among years and farms in terms of the average number of CBB/trap/day, the general pattern of flight was consistent (Fig 3). Seasonal flight patterns include two stages: an initial emergence from January-April which coincides with early fruit development, and a secondary flight which occurs from September-December and coincides with the harvest season (Fig 3). This second flight corresponds to the emergence of new generations of CBB that were the offspring of the initial colonizing females. Although we did not observe any consistent differences in trap capture patterns among farms in the Kona vs. Kaʻu regions, a linear regression revealed a moderate but significant negative correlation between elevation and total trap catch (*R* = -0.48, t = -3.39, p = 0.002).

### Weather variables and CBB flight

**Fig 4:**
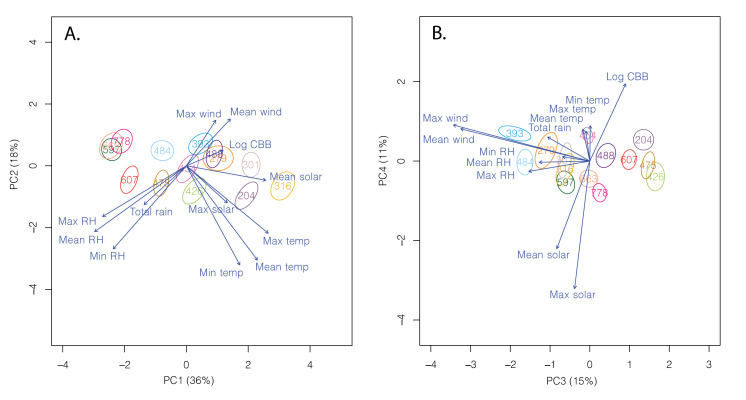
Principal components analysis showing the 95% CI ellipses for each of the 14 study sites labeled by elevation (m). The PCA revealed four significant axes (eigenvalues >1) that explained 78% of the total variation. Arrows represent the direction and strength of the relationship between the 14 sites and each variable.

The PCA revealed four significant axes (eigenvalues >1) that explained 78% of the variation between sites (Table 1). The first PC (36% of variation; Fig 4A) was largely defined by RH, mean and maximum temperature, as well as mean solar radiation; PC2 (18% of variation; Fig 4A) was composed primarily of temperature, and mean and minimum RH; PC3 (15% of variation; Fig 4B) was made up mainly of mean and maximum wind; and PC4 (11% of variation; Fig 4B) was defined by mean and maximum solar radiation and CBB trap catch (Table 1).

**Table 1:** Results from a principal components analysis (PCA) examining environmental variables and CBB trap catch at 14 commercial coffee farms on Hawaii Island. Loadings of these variables are presented for the first four principal components (PCs) which explained 78% of the variation. Significant loadings are highlighted in bold.

Variable	PC1	PC2	PC3	PC4
**Mean Temperature**	**0.342**	**-0.454**	--	0.171
**Minimum Temperature**	0.270	**-0.502**	--	0.191
**Maximum Temperature**	**0.367**	**-0.302**	--	0.161
**Mean RH**	**-0.404**	**-0.290**	-0.230	--
**Minimum RH**	**-0.344**	**-0.391**	-0.125	--
**Maximum RH**	**-0.362**	-0.219	-0.278	--
**Mean Solar**	**0.323**	--	-0.174	**-0.460**
**Maximum Solar**	0.186	-0.169	--	**-0.685**
**Cumulative Rain**	-0.193	-0.177	-0.199	0.113
**Mean Wind**	0.205	0.218	**-0.591**	0.148
**Maximum Wind**	0.145	0.225	**-0.626**	0.166
**CBB catch**	0.154	--	0.187	**0.402**

**Fig 5:**
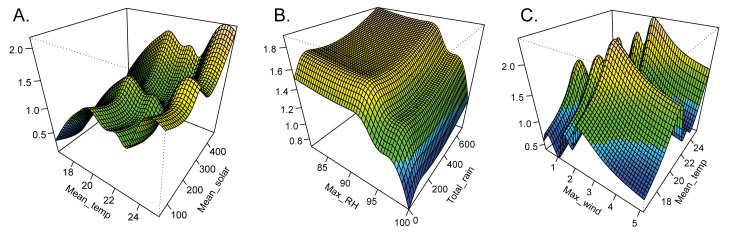
Results from a generalized additive mixed model (GAMM) exploring the effects of mean daily temperature (A, C), mean solar radiation (A), maximum relative humidity (B), cumulative rainfall (B) and maximum wind speed (C) on coffee berry borer flight activity. Three-dimensional contour plots show peaks in CBB flight activity in yellow and decreased activity in cooler colors (green and blue). Note that different scales are used for each x-axis. Units for each of the weather variables are as follows: mean temperature (°C), mean solar radiation (W m^-2^), maximum relative humidity (%), cumulative rainfall (mm), and maximum wind speed (m/s).

The final GAMM explained 75.7% of the deviance (adjusted R^2^ = 0.66, GCV = 0.29), with the following weather variables having a significant relationship with CBB flight at an alpha < 0.05: mean temperature, maximum RH, cumulative rainfall, maximum wind, and mean solar radiation (Table 2). Mean daily temperature had the greatest positive relationship with CBB flight (Table 2) with three peaks observed between 20–26°C (Fig 5A). Mean daily solar radiation also had a positive correlation with CBB flight, with peaks observed at 200–300 W/m^-2^ and 400–500 W/m^-2^ (Fig 5A). Flight levels were generally high at maximum daily RH values between 80–94%, after which they fell sharply (Fig 5B). Cumulative rainfall had a positive correlation with CBB flight up to 100 mm, after which flight decreased (Fig 5B). Flight increased up to maximum daily wind speeds of ~2.5 m/s and subsequently dropped off (Fig 5C). Both the random effects of site (p < 0.001) and year (p = 0.003) also had significant effects on CBB flight (Table 2).

**Table 2:** Results from the generalized additive mixed model (GAMM) analyzing the relationship between five weather variables (fixed effects) plus site and year (random effects) on CBB flight.

Variable	edf	ref.df	F	p-value
**Mean Temperature**	8.432	8.870	5.868	5.07e^-07^***
**Minimum Temperature**	2.138	2.824	1.081	0.282
**Maximum RH**	8.062	8.580	3.504	0.001**
**Minimum RH**	4.075	4.960	1.991	0.096
**Mean Solar**	5.064	5.898	3.234	0.005**
**Cumulative Rain**	7.737	8.355	2.815	0.006**
**Maximum Wind**	8.695	8.948	2.277	0.015*
**Mean Wind**	2.648	3.439	1.693	0.184
**Site**	7.237	8.000	8.520	1.21e^-10^***
**Year**	0.849	1.000	8.834	0.003**

edf–effective degrees of freedom; ref.df–reference degrees of freedom; F–*F*-statistic; p-values were considered significant at the following levels: < 0.001 ‘***’, < 0.01 ‘**’, < 0.05 ‘*’.

## Discussion

Determining the seasonal phenology and abiotic factors involved in flight activity is often a critical step in developing an integrated pest management plan for invasive insects. In the present study we examined 14 commercial coffee farms in two coffee-growing districts on Hawaii Island to elucidate seasonal flight patterns and the correlation of five weather variables with CBB flight as estimated by trap catch. Variation among the three years examined was likely due to a combination of weather events and the timing of study initiation, while variation among farms likely involved differences in management style, timing, and the number of interventions, as well as differences in CBB development times across elevations. Our findings suggest that although there are differences from farm to farm, general patterns of CBB flight activity based on time of year and weather correlates can be described across this variable landscape. We observed two major flight events that were consistent across all three years: an initial emergence from January-April that coincides with early fruit development and a secondary flight that occurs during the harvest season from September-December. We also found that despite the lack of a species-specific lure, trap specificity was generally high with CBB making up 81–93% of the total trap catch across all farms. Farms observed to have the highest percentages of non-CBB beetles in traps were located next to macadamia nut orchards (hosts for tropical nut borer) or forests (hosts for black twig borer) or had other fruit and nut trees (hosts for *Cryphalus* sp.) interplanted with the coffee.

These results agree with findings from two earlier studies that examined CBB flight patterns on Hawaii Island. Messing [20] reported CBB flight from November-March at two farms in Kona. That study also found that 1:1 and 3:1 ratios of methanol:ethanol captured similar numbers of beetles, and that non-target beetles made up an insignificant proportion of trap catch relative to CBB (3–7 non-target beetles/trap/day compared to 100–400 CBB/trap/day). Our findings are also in line with those of Aristizábal et al. [21], who examined flight activity at 15 farms in Kona and Kaʻu using 3:1 methanol:ethanol baited funnel traps. That study reported a small peak in flight from May-July and a larger peak from December-February. The authors suggested that peaks appeared to coincide with increased levels of rainfall following a dry period, although weather data was not available for each farm, excluding statistical analyses. Aristizábal et al. [21] also estimated that non-target insects made up < 5% of all trap catch based on subsampling from a few farms.

Seven of the 10 variables included in the GAMM were found to have a significant relationship with CBB flight activity. Of the eight fixed effects, mean daily temperature, mean solar radiation, maximum RH, maximum wind speed and cumulative rainfall were significant, while both the random effects of site and year also had a significant correlation with flight. That the best model explained only 75.7% of the observed variance likely reflects the absence of additional weather variables that were not measured (e.g., barometric pressure), as well as variation that was missed due to sampling frequency.

Mean daily air temperature was observed to be the single weather variable with the strongest (positive) correlation with CBB flight activity across all sites. This was also reflected in the PCA, where together with RH and mean solar radiation, mean and maximum temperature accounted for most of the variance in the first component. This is relatively unsurprising since insects are poikilotherms, meaning their body temperature depends on ambient environmental temperature. Many aspects of insect biology are driven by temperature including generation length, rate of development, mating activity and dispersal [22].

Chen and Seybold [23] reported a lower threshold of 11°C, an optimum of 27°C and an upper threshold of 39°C for flight of the walnut twig beetle (*Pityophthorus juglandis* Blackman). In a controlled laboratory setting with RH held at 90% and 100%, Baker et al. [24] reported low CBB emergence from dried berries at temperatures below 20°C, a marked increase in emergence from 20–25°C, and no significant increase above 25°C. In the present study under highly variable field conditions, we observed that most flight events took place when mean daily temperatures were between 20–26°C, with very few events below 16°C or above 32°C.

Along with a positive correlation to increasing temperature, we found a positive significant relationship between CBB flight and mean daily solar radiation. This is line with the findings of several studies that reported temperature and solar radiation as the main abiotic factors positively influencing beetle flight [23, 25–27]. We also observed a significant negative correlation between elevation and total CBB capture. Hamilton et al. [28] showed that CBB development on Hawaii Island is faster at low elevations primarily due to higher temperatures. The authors estimated 4–5 generations per season at low elevations (200–300 m), compared to 2–3 generations per season at high elevations (600–800 m). Thus, the higher abundance of CBB caught at lower elevations may be related to the shorter development times driven by temperature at these sites.

In accordance with numerous studies on insect flight, we observed a significant positive relationship between maximum daily relative humidity until values reached ~94%, after which flight activity declined. CBB may not leave the berries during periods of very high RH as this may indicate rain (along with an associated drop in barometric pressure, not measured in the current study), causing them to shelter. In addition, greater CBB mortality can occur during periods of high RH due to proliferation of *B*. *bassiana* under moist, humid conditions. Lastly, at very high RH there is a higher requirement of wing-beat frequency, which is metabolically costly [29, 30].

In contrast to our findings, under laboratory conditions with temperature held at 25°C, Baker et al. [24] described high CBB emergence from infested two-month-old berries at RH values of 20% and 55%, minimum emergence at 78% and 90%, and a steady increase in emergence from 94–100%. It is likely that differences between this study and our findings are related to the setting under which emergence was estimated. We did not observe a minimum RH lower than 52%, and mean RH values ranged from 80–90% for most sites/years. Maximum RH values were typically >94% through spring and summer and dropped during the winter months (November-March), which coincided with peak CBB flight.

We observed a similar trend for maximum wind speed, with a positive correlation between flight and wind speeds up to ~2.5 m/s and then a negative relationship with wind speeds above that value. Chen and Seybold [23] reported that *P*. *juglandis* flight was limited at very low wind speeds and peaked when temperature was ~30°C and wind speed was 2 km/h. Pawson et al. [31] reported low flight activity of the bark beetle *Hylurgus ligniperda* Fabricius at very low wind speeds, an increase with rising wind speeds, and a peak at 2 m/s. Thus, some wind may help to initiate flight as well as provide olfactory cues to allow detection of resources, but at very high wind speeds flight may be inhibited. The long-distance dispersal of many smaller insects is dictated mostly by wind [32–34]; CBB are weak flyers that tend to hover near coffee plants at a height of 1–2 m (M. Johnson, unpub. data) where ambient wind speeds are generally low [35], which may suggest relatively infrequent long-distance dispersal events. Lastly, cumulative rainfall had a positive relationship with CBB flight up to a point; flight appeared to be inhibited during periods of heavy rainfall (>100 mm). Other studies have reported negative effects of heavy rainfall on bark beetle flight [36–38]; the very small size of CBB likely precludes its movement during periods of high rainfall.

It is difficult to disentangle the individual correlations with weather and flight discussed above from the effect of the season on coffee phenology, but even without direct evidence of causation, the pattern of CBB movement in Hawaii coffee plantations comes into focus. At the very beginning of the year the new coffee crop must become mature enough (>20% dry matter content [39, 40]) to be infested by the previous season’s beetles. These CBB are mostly in raisins (dried berries) on the ground under the coffee trees or on the raisins remaining on the branches–the latter being the most heavily infested repositories on a per-bean basis [41]. The first flight suggests movement from these refugia into developing green berries in the first quarter of the calendar year. It is possible that the first flight is triggered by rainfall events that occur early in the year, which initiates coffee plant flowering after a period of drought and dormancy. Rain and increases in RH have been reported to cause CBB to emerge from raisins [21, 24]. Baker et al. [24] inferred that this could be an escape response due to moist conditions that favor entomopathogenic fungi, and/or CBB may prefer to initiate flight with some humidity in the air to prevent dessication while seeking new berries to infest [24]. Given that few berries may be available early in the season, CBB will have to travel more extensively to find suitable hosts. Once a suitable berry is located, the female CBB will penetrate the exocarp and wait to complete entry into the coffee bean until conditions are suitable, or may begin boring immediately into the seed depending on fruit stage [1].

After the first flight, CBB begin reproducing within the beans and berry development continues. During this middle part of the year, there is very little baseline movement of CBB and trap catches are low. During this period, host berries are abundant. RH and rainfall are typically high from June-August on Hawaii Island, and this may limit CBB flight as well as increase mortality. The second major flight, observed here in the last quarter of the calendar year, is likely driven by waning food supplies in the original berries in combination with physical disturbance during harvesting, strip-picking and tree pruning. The high temperatures that characterize coffee plantations on Hawaii Island from August-September contribute to shortened CBB development times [28]. As the numbers of CBB increase in the individual beans, food is reduced and movement again becomes necessary. Based on observations of lower flight activity in this second part of the year in feral and unmanaged sites vs. well-managed sites on Hawaii Island [42], physical disturbance via management practices are a larger stimulus for this second flight relative to the need to locate food, which is likely the primary stimulus for the first flight of the season.

Given the large travel distance between sites, all traps were sampled on a bi-weekly schedule, such that correlating weather events to trap catch at finer scales was not possible. Future studies that increase sampling frequency to weekly or daily time scales will be needed to further characterize the precise conditions that trigger mass CBB flight events. The timing of daily CBB flight and the weather events correlated with flight activity on an hourly basis are currently being examined at several sites on Hawaii Island. In addition, CBB flight sensors are being developed and tested under field conditions in an effort to better capture mass flight events in real-time, and limit the reliance on traps as a proxy for flight. Ultimately, tying in flight sensors with other precision agriculture technology such as mobile applications for pest monitoring will provide growers with the IPM tools necessary to properly manage this global pest of coffee. We expect that predictive models coupled with sensor technology and crop monitoring applications will become increasingly important as climate change is predicted to increase pest and disease pressures across many coffee growing regions of the world.

## Conclusions

In the present study we found that CBB flight on Hawaii Island has a significant positive relationship with temperature and solar radiation. We also found a significant positive correlation between flight and RH, rainfall and wind speed up to a certain threshold, after which flight decreased. Our investigation into CBB flight activity revealed important insights that will be useful for the development of future flight prediction models based on weather data and time of year. The simplest models would relate calendar date to expected flight, but the observed correlations between individual variables and trap catch could be used to predict the exact timing of flights especially due to weather anomalies.
